# Human milk oligosaccharide composition following supplementation with folic acid vs (6*S*)-5-methyltetrahydrofolic acid during pregnancy and mediation by human milk folate forms

**DOI:** 10.1038/s41430-023-01376-7

**Published:** 2023-12-06

**Authors:** Kelsey M. Cochrane, Jeffrey N. Bone, Crystal D. Karakochuk, Lars Bode

**Affiliations:** 1 Food, Nutrition, and Health, Faculty of Land and Food Systems, University of British Columbia, Vancouver, BC Canada; 2 BC Children’s Hospital Research Institute, Vancouver, BC Canada; 3 Obstetrics and Gynaecology, Faculty of Medicine, University of British Columbia, Vancouver, BC Canada; 4 Department of Pediatrics, Larsson Rosenquist Foundation Mother-Milk-Infant Center of Research Excellence (MOMI CORE), and Human Milk Institute (HMI), University of California San Diego, La Jolla, CA USA

**Keywords:** Biomarkers, Metabolism

## Abstract

Supplementation with folic acid versus (6*S*)-5-methyltetrahydrofolic acid (5-MTHF) results in different folate forms in human milk, with folic acid increasing unmetabolized folic acid (UMFA) at the expense of reduced folate forms. It is unknown whether folate forms present in human milk have further effects on human milk composition, such as human milk oligosaccharide (HMO) concentrations. We randomized 60 pregnant women in Canada to 0.6 mg/day folic acid or (6*S*)-5-MTHF. Human milk folate forms (LC-MS/MS) and nineteen HMOs (HPLC) were quantified at 1 week postpartum. Linear regression and causal mediation analysis were used to evaluate the effect of folate supplementation on HMO concentrations, and possible mediation by concentrations of UMFA and reduced folate forms in human milk (controlling for secretor status and parity). HMO concentrations were not different between groups, with no evidence of mediation by reduced folate forms; however, increased UMFA was associated with reduced concentrations of total HMOs and 3’-sialyllactose.

## Introduction

Human milk oligosaccharides (HMOs) are a group of complex carbohydrates that are highly abundant in human milk and play an important role in supporting infant health [1]. More than 150 different HMO structures have been identified and HMO composition can vary significantly between individuals and over the course of lactation [2]. This variation is driven by both fixed and modifiable factors, including maternal nutrition and supplementation [2, 3]. For example, multiple micronutrient supplementation has been associated with HMO composition [3], but little is known regarding the effect of individual micronutrients.

North American guidelines recommend folic acid supplementation throughout pregnancy and lactation to support optimal growth and development. Folic acid is a synthetic folate form that must be reduced for use in the body; capacity for this is limited, resulting in unmetabolized folic acid (UMFA) [4], a biologically inactive form. Reduced folates serve as co-factors in one carbon metabolism, re-methylating homocysteine to methionine and producing numerous outputs including *S*-adenosylmethionine, the universal methyl donor [4].

There is an increasing interest in supplementation with (6*S*)-5-methyltetrahydrofolic acid (5-MTHF) as an alternative to folic acid, as this form is reduced and not metabolically limited [4, 5]. We previously reported that supplementation with folic acid altered folate forms present in human milk as compared to (6*S*)-5-MTHF, increasing the proportion of human milk UMFA by 14-fold, seemingly at the expense of reduced folate forms [6]. It is unknown whether maternal exposure to folic acid supplementation, resulting in higher UMFA in human milk, has further downstream effects on human milk composition. Thus, our aim was to evaluate the effect of supplementation with folic acid versus (6*S*)-5-MTHF on HMO composition, and mediation of this effect by folate forms present in human milk.

## Methods

The full trial protocol is published elsewhere [7] and is registered at ClinicalTrials.gov (NCT04022135). Pregnant women (*n* = 60) in Vancouver, Canada were recruited via printed posters and digital advertising, and randomized to 0.6 mg/day folic acid or an equimolar dose (0.625 mg/day) of (6*S*)-5-MTHF at 8–21 weeks’ gestation. Informed consent was obtained, and participants were supplemented for 16 weeks of pregnancy (starting at 8–21 weeks gestation); after 16 weeks, participants had the option to provide separate informed consent to continue supplementation until ~1 week postpartum. At ~1-week postpartum, *n* = 42 provided a human milk specimen (see detailed collection methods elsewhere [7]) for quantification of folate forms, including UMFA, tetrahydrofolate (THF), 5-methylTHF, 5,10-methenylTHF, 5-formylTHF, and 4α-hydroxy-5-methylTHF via LC-MS/MS, and nineteen HMOs via HPLC with fluorescence detection [6, 8]. Quantification of maternal red blood cell and serum folate concentrations via microbiological assay and genotyping of *MTHFR* 677C>T was conducted as part of a separate investigation [9].

Multivariable linear regression (or quantile regression for non-normally distributed HMOs) was used to evaluate the difference in HMO concentrations between groups and the association of HMO concentrations with human milk UMFA. Mediation analysis (R package: CMAverse; methods of Valeri and Vanderweele) was used to evaluate the effect of folate supplementation on HMO concentrations, and whether the effect was mediated by human milk folate forms; the two mediators included concentrations of human milk UMFA and reduced folate forms [10]. Natural direct and indirect effects (e.g., intervention effects not mediated by, and mediated by, human milk folate forms, respectively) were estimated. All analyses were conducted on an intention-to-treat basis and adjusted for secretor status (2’FL ≥ 100 nmol/mL) and parity.

## Results

Mean ± SD age of participants was 33 ± 3 years; they were predominantly of European ethnicity (55%) with post-secondary education (98%). In those supplemented with (6*S*)-5-MTHF (*n* = 22) and folic acid (*n* = 20), respectively, *n* = 18 (82%) and *n* = 11 (55%) were nulliparous, and *n* = 16 (73%) and *n* = 13 (65%) were identified as secretors. No significant differences in maternal folate status were found between groups or genotypes, with no occurrences of folate deficiency throughout pregnancy or at 1-week postpartum [9]. Of note, the homozygous (TT) polymorphism was only present in *n* = 2 participants in the folic acid group, limiting our ability to interpret its effect [9]. Total human milk folate concentrations were not different between groups [6]; in the (6*S*)-5-MTHF and folic acid groups, respectively, the mean ± SD proportion of UMFA as part of total human milk folate was: 2 ± 2% and 29 ± 14% and the proportion of reduced folate forms was: 98 ± 2% and 71 ± 14%.

**Fig. 1:**
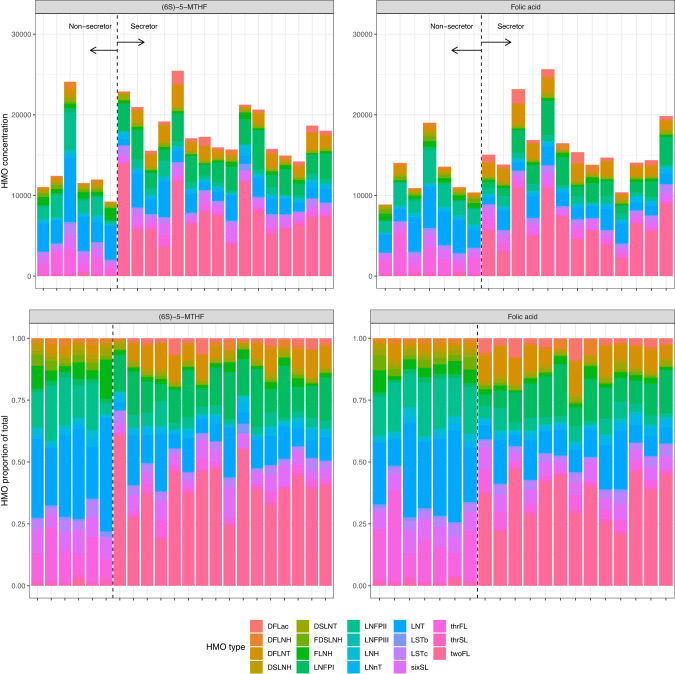
HMO profiles following supplementation with (6S)-5-MTHF (*n* = 22) versus folic acid (*n* = 20). Each column represents an individual participant, sorted by secretor status in each intervention group. Total HMO concentrations (nmol/mL) and the relative abundance of each HMO (proportion of total HMO) are presented. HMO Human milk oligosaccharides, DFLac difucosyllactose, DFLNH difucosyllacto-N-hexaose, DFLNT difucosyllacto-N-tetrose, DSLNH disialyllacto-N-hexaose, DSLNT disialyllacto-N-tetraose, FDSLNH fucodisiayllacto-N-hexaose, FLNH fucosyllacto-N-hexaose, LNFP I lacto-N-fucopentaose I, LNFP II lacto-N-fucopentaose II, LNFP III lacto-N-fucopentaose III, LNH lacto-N-hexaose, LNnT lacto-N-neotetraose, LNT lacto-N-tetraose, LSTb sialyl-lacto-N-tetraose b, LSTc sialyl-lacto-N-tetraose c, sixSL 6’-sialyllactose, thrFL 3-fucosyllactose, thrSL 3’-sialyllactose, twoFL 2’-fucosyllactose.

HMO profiles of secretors and non-secretors in each intervention group are presented in Fig. 1. Overall, HMO concentrations exhibited a high degree of inter-individual variability and direction of the effect was uncertain between intervention groups (Supplementary Table 1). The results of the mediation analyses are presented in Table 1. The percent of the intervention effect mediated by concentrations of reduced folate forms was nearly zero for all HMOs, with very tight 95% CIs, suggesting little evidence of mediation. A high degree of variability was observed for mediation by concentrations of human milk UMFA, limiting substantive conclusions for its effect. However, increased UMFA in human milk (nmol/L) was associated with reduced concentrations of total HMOs (β-coefficient: −139; 95% CI: −258 to −20 nmol/mL) and the individual HMO 3’-sialyllactose (β-coefficient: −1.7; 95% CI: −3.0 to −0.4 nmol/mL).

**Table 1:** Effect of folate supplementation on HMO composition and mediation by human milk folate forms.

HMO nmol/mL	Mediator nmol/L	^a^Total effect coefficient (95% CI)	Pure natural direct effect coefficient (95% CI)	Total natural indirect effect coefficient (95% CI)	^b^Percent mediation % (95% CI)
2’-fucosyllactose (2’FL)	UMFA	1142 (−808, 3091)	−5305 (−20311, 9702)	6447 (−9214, 22107)	5.6 (−7.7, 19)
	Reduced folate forms	932 (−615, 2479)	927 (−620, 2474)	4.9 (−85, 95)	0 (−0.1, 0.1)
3-fucosyllactose (3FL)	UMFA	−139 (−649, 371)	−671 (−5165, 3823)	532 (−4147, 5211)	−3.8 (−45, 38)
	Reduced folate forms	−185 (−641, 270)	−183 (−644, 278)	−2.2 (−34, 29)	0 (−0.2, 0.2)
Difucosyllactose (DFLac)	UMFA	−96 (−392, 199)	744 (−1646, 3133)	−840 (−3330, 1650)	8.7 (−20, 38)
	Reduced folate forms	−44 (−288, 200)	−48 (−295, 199)	3.5 (−38, 45)	−0.1 (−1.1, 1)
3’-sialyllactose (3’SL)	UMFA	21 (−13, 55)	−87 (−341, 167)	108 (−157, 373)	5.2 (−7, 17)
	Reduced folate forms	18 (−10, 46)	18 (−8.6, 45)	−0.8 (−9.6, 8.1)	0 (−0.6, 0.5)
6’-sialyllactose (6’SL)	UMFA	181 (−35, 397)	112 (−1821, 2044)	70 (−1941, 2081)	0.4 (−11, 11)
	Reduced folate forms	195 (−3.1, 394)	191 (−11, 393)	4.6 (−49, 58)	0 (−0.2, 0.3)
Lacto-N-tetraose (LNT)	UMFA	622 (−273, 1517)	−1291 (−8899, 6317)	1913 (−6010, 9837)	3.1 (−8.6, 15)
	Reduced folate forms	527 (−247, 1301)	541.5 (−218, 1301)	−14.5 (−184, 155)	0 (−0.4, 0.3)
Lacto-N-neotetraose (LNnT)	UMFA	60 (−109, 229)	−754 (−1768, 260)	814 (−250, 1878)	14 (−22, 50)
	Reduced folate forms	16 (−92, 123)	21 (−88, 131)	−5.8 (−72, 61)	−0.4 (−5.9, 5.1)
Lacto-N-fucopentaose I (LNFP I)	UMFA	678 (−504, 1859)	−5875 (−11357, −393)	6553 (746, 12360)	9.7 (−6.2, 26)
	Reduced folate forms	401 (−191, 994)	415 (−160, 989)	−13 (−167, 140)	0 (−0.4, 0.4)
Lacto-N-fucopentaose II (LNFP II)	UMFA	4.2 (−523, 532)	−736 (−5350, 3877)	740 (−4064, 5544)	–
	Reduced folate forms	−44 (−518, 429)	−33 (−515, 448)	−11 (−140, 118)	0.2 (−3.5, 4)
Lacto-N-fucopentaose III (LNFP III)	UMFA	15 (−11, 41)	−88 (−265, 90)	103 (−83, 289)	6.8 (−6.5, 20)
	Reduced folate forms	12 (−7.1, 30)	12 (−6.8, 30)	−0.1 (−1.9, 1.6)	0 (−0.2, 0.1)
Sialyl-lacto-N-tetraose b (LSTb)	UMFA	71 (−55, 197)	−527 (−1299, 245)	599 (−211, 1408)	8.4 (−6.7, 24)
	Reduced folate forms	43 (−37, 123)	45 (−35, 124)	−1.6 (−20, 17)	0 (−0.5, 0.4)
Sialyl-lacto-N-tetraose c (LSTc)	UMFA	82 (−145, 309)	−812 (−2433, 810)	893 (−801, 2587)	11 (−19, 41)
	Reduced folate forms	36 (−132, 204)	42 (−123, 207)	−6.1 (−76, 64)	−0.2 (−2.5, 2.1)
Difucosyllacto-N-tetrose (DFLNT)	UMFA	−127 (−599, 344)	553 (−3567, 4672)	−680 (−4968, 3608)	5.3 (−26, 37)
	Reduced folate forms	−106 (−530, 317)	−99 (−528, 330)	−7.1 (−91, 76)	0.1 (−0.8, 0.9)
Lacto-N-hexaose (LNH)	UMFA	−2.9 (−65, 60)	151 (−369, 670)	−153 (−695, 388)	–
	Reduced folate forms	3.5 (−50, 57)	3.7 (−50, 57)	−0.3 (−4.1, 3.6)	−0.1 (−1.7, 1.5)
Disialyllacto-N-tetraose (DSLNT)	UMFA	64 (−61, 188)	−584 (−1242, 74)	648 (−46, 1341)	10 (−8.7, 29)
	Reduced folate forms	38 (−32, 108)	39 (−30, 108)	−0.8 (−11, 9.4)	0 (−0.3, 0.3)
Fucosyllacto-N-hexaose (FLNH)	UMFA	123 (−27, 272)	140 (−1190, 1469)	−17 (−1401, 1367)	−0.1 (−12, 11)
	Reduced folate forms	124 (−14, 263)	123 (−16, 263)	1.2 (−14, 16)	0 (−0.1, 0.1)
Difucosyllacto-N-hexaose (DFLNH)	UMFA	73 (−62, 207)	−241 (−1374, 892)	313 (−867, 1494)	4.3 (−11, 19)
	Reduced folate forms	59 (−57, 174)	59 (−55, 174)	−0.8 (−12, 10)	0 (−0.2, 0.2)
Fucodisiayllacto-N-hexaose (FDSLNH)	UMFA	−59 (−151, 34)	399 (−145, 943)	−458 (−1028, 113)	7.8 (−4.9, 21)
	Reduced folate forms	−37 (−94, 20)	−38 (−94, 17)	1.3 (−14, 17)	0 (−0.5, 0.4)
Disialyllacto-N-hexaose (DSLNH)	UMFA	26 (−39, 92)	303 (−147, 753)	−276 (−747, 194)	−11 (−47, 26)
	Reduced folate forms	40 (−6.5, 86)	39 (−7, 85)	1.2 (−13, 15)	0 (−0.3, 0.4)

All effects are adjusted for secretor status and parity.

*–* could not be estimated, *HMO* human milk oligosaccharide, *UMFA* unmetabolized folic acid.

^a^The *total effect* equals the sum of direct and indirect effects;

^b^Percent of the intervention effect mediated by human milk folate forms.

## Discussion

The effect of folic acid versus (6*S*)-5-MTHF supplementation on HMO concentrations remains uncertain in this cohort of Canadian women, given high variability in our results. However, we observed no evidence of effect mediation based on concentrations of reduced folate forms in human milk. It remains possible that there is a threshold for folate in human milk required to alter HMO synthesis; perhaps *sufficient* reduced folate was present across groups, despite a lower proportion in those supplementing with folic acid. Possible mediation due to human milk UMFA is less clear; perhaps this is due to the very low concentrations following (6*S*)-5-MTHF supplementation, limiting sensitivity to observe an effect. Further, although the exposure was randomized, unmeasured confounding between mediators and outcomes remains possible and may affect results. Ultimately, higher milk UMFA was associated with reduced concentrations of total HMOs and the individual HMO 3’-sialyllactose, which has been associated with inflammation and infection risks [11]. While factors other than the study intervention may have contributed to human milk UMFA concentrations (e.g., diet), we speculate that folic acid supplementation is the most important contributor [6].

Perhaps the lack of association found between folic acid supplementation and HMO concentrations, mediated by increased human milk UMFA, was due to the small sample size; particularly, given our observation of decreased HMOs with increased human milk UMFA. Further research in a larger cohort is warranted and should utilize a longitudinal approach to evaluate temporal changes in HMOs across lactation. Should future research confirm an association between folic acid supplementation or UMFA and HMO concentrations, ascertaining underlying mechanisms is critical. To our knowledge, this is not described in current literature. Whether exposure to UMFA elicits negative biological or clinical effects remains widely debated, but any risk is proposed to increase with increasing exposure [12]. This study is one step towards understanding how modifiable factors impact HMOs; furthering this understanding can support infant health, as it may enable targeted interventions that can shift human milk composition towards a more favorable profile.

### Supplementary information

Multivariable linear/quantile regression to evaluate the difference in HMO concentrations between intervention groups and by concentrations of human milk UMFA

## Data Availability

The datasets generated during and/or analyzed during the current study are available from the corresponding author on reasonable request.
