# Supplementary material for: Human milk oligosaccharide composition following supplementation with folic acid vs (6S)-5-methyltetrahydrofolic acid during pregnancy and mediation by human milk folate forms
Source: Eur J Clin Nutr. 2023 Dec 6;78(4):351–5. doi: 10.1038/s41430-023-01376-7 (PMC11003863; doi:10.1038/s41430-023-01376-7)
Supplement: Supplementary file 1 — Multivariable linear/quantile regression to evaluate the difference in HMO concentrations between intervention groups and by concentrations of human milk UMFA [file 41430_2023_1376_MOESM1_ESM.pdf]

Supplemental Table 1: Multivariable linear/quantile regression to evaluate the difference in HMO concentrations between intervention groups and by concentrations of human milk UMFA

HMO (nmol/mL)	**Difference between intervention groups**<br>β-coefficient (95% CI)	**Difference by milk UMFA concentrations (nmol/L)**<br>β-coefficient (95% CI)	**(6S)-5-MTHF (*n* =22);** crude concentrations (nmol/mL)<br>Median, IQR (and mean ± SD if normal distribution)	**Folic acid (*n* =20);** crude concentrations (nmol/mL)<br>Median, IQR (and mean ± SD if normal distribution)
**HMO sum**			**16519 (14210, 20648); 16,990 ± 4380**	**14217 (12294, 16665); 15,066 ± 4266**
**Folate form ([6S)]-5-MTHF as the reference group)**	**-2077 (-4557, 403); p=0.098**			
**Human milk UMFA (nmol/L)**		**-139 (-258, -20); p=0.023**		
Secretor status (non-secretor as the reference group)	4576 (1997, 7156); p=0.001	4833 (2336, 7330); p=0.000		
parity (multiparous as the reference group)	-1891 (-4577, 795); p=0.162	-1280 (-3775, 1214); p=0.305		
**2'-fucosyllactose (2'FL)**			**5765 (279, 7469)**	**4115 (58, 6003)**
**Folate form ([6S)]-5-MTHF as the reference group)**	**-12 (-1296, 1272); p=0.985**			
**Human milk UMFA (nmol/L)**		**-18 (-81, 44); p=0.559**		
Secretor status (non-secretor as the reference group)	6221 (4886, 7557); p=0.000	6134 (4823, 7445); p=0.000		
parity (multiparous as the reference group)	7.8 (-1383, 1398); p=0.991	144 (-1165, 1454); p=0.825		
**3-fucosyllactose (3FL)**			**544 (300, 1031)**	**824 (492, 1560)**
**Folate form ([6S)]-5-MTHF as the reference group)**	**87 (-440, 614); p=0.739**			
**Human milk UMFA (nmol/L)**		**0.3 (-24, 25); p=0.982**		
Secretor status (non-secretor as the reference group)	-1470 (-2019, -922); p=0.000	-1482 (-1994, -970); p=0.000		
parity (multiparous as the reference group)	-199 (-770, 371); p=0.484	-126 (-637, 386); p=0.621		
**difucosyllactose (DFLac)**			**236 (47, 394)**	**255 (16, 502)**
**Folate form ([6S)]-5-MTHF as the reference group)**	**22 (-161, 204); p=0.809**			
**Human milk UMFA (nmol/L)**		**1.7 (-7, 10); p=0.700**		
Secretor status (non-secretor as the reference group)	331 (141, 521); p=0.001	325 (140, 509); p=0.001		
parity (multiparous as the reference group)	-2.5 (-200, 195); p=0.979	-9.8 (-194, 175); p=0.915		
**3'-sialyllactose (3'SL)**			**190 (153, 214); 189 ± 43**	**177 (145, 215); 182 ± 55**
**Folate form ([6S)]-5-MTHF as the reference group)**	**-18 (-46, 10); p=0.201**			
**Human milk UMFA (nmol/L)**		**-1.7 (-3, -0.4); p=0.014**		
Secretor status (non-secretor as the reference group)	-47 (-76, -18); p=0.002	-45 (-72, -17); p=0.002		
parity (multiparous as the reference group)	-25 (-55, 5.3); p=0.103	-20 (-47, 7.6); p=0.152		
**6'-sialyllactose (6'SL)**			**998 (754, 1187)**	**846 (704, 1061)**
**Folate form ([6S)]-5-MTHF as the reference group)**	**-188 (-505, 129); p=0.238**			
**Human milk UMFA (nmol/L)**		**-11 (-26, 4.4); p=0.157**		
Secretor status (non-secretor as the reference group)	20 (-310, 350); p=0.902	87 (-237, 411); p=0.589		
parity (multiparous as the reference group)	-240 (-583, 103); p=0.165	-175 (-499, 149); p=0.281		
**lacto-N-tetraose (LNT)**			**2115 (1322, 3499)**	**2237 (1345, 2661)**
**Folate form ([6S)]-5-MTHF as the reference group)**	**-241 (-1252, 770); p=0.632**			
**Human milk UMFA (nmol/L)**		**-12 (-63, 39); p=0.634**		
Secretor status (non-secretor as the reference group)	-2500 (-3552, -1449); p=0.000	-2584 (-3645, -1524); p=0.000		
parity (multiparous as the reference group)	-79 (-1175, 1016); p=0.884	7 (-1052, 1066); p=0.989		
**lacto-N-neotetraose (LNnT)**			**378 (213, 476); 377 ± 217**	**303 (244, 417); 313 ± 126**
**Folate form ([6S)]-5-MTHF as the reference group)**	**-26 (-132, 80); p=0.622**			
**Human milk UMFA (nmol/L)**		**-2 (-7.6, 2.8); p=0.358**		
Secretor status (non-secretor as the reference group)	144 (34, 255); p=0.012	148 (39, 258); p=0.009		
parity (multiparous as the reference group)	99 (-16, 214); p=0.090	106 (-2.9, 216); p=0.056		
**lacto-N-fucopentaose I (LNFP I)**			**2150 (527, 3171)**	**1078 (231, 2349)**
**Folate form ([6S)]-5-MTHF as the reference group)**	**-142 (-1011, 727); p=0.742**			

**Human milk UMFA (nmol/L)**		**-16 (-60, 29); p=0.477**		
Secretor status (non-secretor as the reference group)	2172 (1268, 3076); p=0.000	2160 (1223, 3096); p=0.000		
parity (multiparous as the reference group)	-10 (-951, 931); p=0.983	-18 (-954, 917); p=0.968		
**lacto-N-fucopentaose II (LNFP II)**			**671 (383, 1477)**	**878 (599, 1578)**
**Folate form ([6S)]-5-MTHF as the reference group)**	**-77 (-524, 371); p=0.731**			
**Human milk UMFA (nmol/L)**		**-1.9 (-24, 20); p=0.862**		
Secretor status (non-secretor as the reference group)	-1415 (-1880, -950); p=0.000	-1351 (-1818, -884); p=0.000		
parity (multiparous as the reference group)	-209 (-693, 276); p=0.388	-148 (-615, 318); p=0.523		
**lacto-N-fucopentaose III (LNFP III)**			**52 (40, 80)**	**50 (31, 67)**
**Folate form ([6S)]-5-MTHF as the reference group)**	**6.4 (-24, 37); p=0.674**			
**Human milk UMFA (nmol/L)**		**-0.7 (-2, 0.7); p=0.317**		
Secretor status (non-secretor as the reference group)	-5.9 (-38, 26); p=0.709	-4.1 (-33, 24); p=0.773		
parity (multiparous as the reference group)	12 (-21, 45); p=0.478	3 (-26, 32); p=0.833		
**sialyl-lacto-N-tetraose b (LSTb)**			**97 (79, 112)**	**102 (71, 119)**
**Folate form ([6S)]-5-MTHF as the reference group)**	**0.1 (-33, 33); p=0.995**			
**Human milk UMFA (nmol/L)**		**-1 (-2.7, 0.6); p=0.216**		
Secretor status (non-secretor as the reference group)	-23 (-57, 12); p=0.189	-17 (-52, 18); p=0.335		
parity (multiparous as the reference group)	-15 (-51, 20); p=0.394	-27 (-62, 7.7); p=0.122		
**sialyl-lacto-N-tetraose c (LSTc)**			**462 (357, 634)**	**473 (366, 580)**
**Folate form ([6S)]-5-MTHF as the reference group)**	**2.6 (-194, 199); p=0.979**			
**Human milk UMFA (nmol/L)**		**0.1 (-7.9, 8.2); p=0.970**		
Secretor status (non-secretor as the reference group)	101 (-104, 306); p=0.326	100 (-70, 269); p=0.240		
parity (multiparous as the reference group)	-11 (-225, 202); p=0.914	-13 (-182, 156); p=0.880		
**difucosyllacto-N-tetrose (DFLNT)**			**906 (111, 1408)**	**967 (243, 1569)**
**Folate form ([6S)]-5-MTHF as the reference group)**	**138 (-432, 708); p=0.627**			
**Human milk UMFA (nmol/L)**		**-7.7 (-37, 21); p=0.596**		
Secretor status (non-secretor as the reference group)	905 (312, 1498); p=0.004	983 (372, 1594); p=0.002		
parity (multiparous as the reference group)	-328 (-945, 289); p=0.288	-268 (-879, 342); p=0.379		
**lacto-N-hexaose (LNH)**			**164 (125, 240)**	**190 (158, 248)**
**Folate form ([6S)]-5-MTHF as the reference group)**	**19 (-49, 87); p=0.575**			
**Human milk UMFA (nmol/L)**		**1.1 (-2.1, 4.3); p=0.503**		
Secretor status (non-secretor as the reference group)	-91 (-162, -20); p=0.014	-92 (-159, -25); p=0.009		
parity (multiparous as the reference group)	-10 (-84, 64); p=0.777	-18 (-85, 49); p=0.595		
**disialyllacto-N-tetraose (DSLNT)**			**257 (212, 338)**	**249 (182, 300)**
**Folate form ([6S)]-5-MTHF as the reference group)**	**-21 (-117, 74); p=0.652**			
**Human milk UMFA (nmol/L)**		**-2.3 (-7.1, 2.6); p=0.349**		
Secretor status (non-secretor as the reference group)	23 (-76, 123); p=0.635	11 (-91, 112); p=0.830		
parity (multiparous as the reference group)	-28 (-131, 76); p=0.593	-21 (-123, 80); p=0.674		
**fucosyllacto-N-hexaose (FLNH)**			**413 (281, 549)**	**350 (247, 554)**
**Folate form ([6S)]-5-MTHF as the reference group)**	**-40 (-220, 140); p=0.653**			
**Human milk UMFA (nmol/L)**		**1 (-7.8, 9.9); p=0.819**		
Secretor status (non-secretor as the reference group)	-262 (-449, -75); p=0.007	-252 (-439, -67); p=0.009		
parity (multiparous as the reference group)	-7.6 (-203, 187); p=0.937	0.9 (-185, 187); p=0.993		
**difucosyllacto-N-hexaose (DFLNH)**			**212 (145, 271)**	**169 (114, 284)**
**Folate form ([6S)]-5-MTHF as the reference group)**	**-83 (-213, 47); p=0.204**			
**Human milk UMFA (nmol/L)**		**-3.1 (-9.6, 3.5); p=0.351**		
Secretor status (non-secretor as the reference group)	-76 (-211, 59); p=0.263	-43 (-180, 95); p=0.534		
parity (multiparous as the reference group)	-58 (-198, 83); p=0.413	-7.3 (-145, 130); p=0.915		
**fucodisiayllacto-N-hexaose (FDSLNH)**			**86 (62, 200)**	**136 (82, 299)**
**Folate form ([6S)]-5-MTHF as the reference group)**	**14 (-64, 91); p=0.721**			
**Human milk UMFA (nmol/L)**		**1 (-2.8, 4.7); p=0.610**		

Secretor status (non-secretor as the reference group)	-275 (-355, -194); p=0.000	-275 (-354, -197); p=0.000		
parity (multiparous as the reference group)	1.9 (-82, 86); p=0.964	-0.7 (-79, 78); p=0.986		
**disialyllacto-N-hexaose (DSLNH)**			**194 (168, 268)**	**183 (147, 218)**
**Folate form ([6S)]-5-MTHF as the reference group)**	**-45 (-104, 14); p=0.128**			
**Human milk UMFA (nmol/L)**		**-1.4 (-4.9, 2); p=0.407**		
Secretor status (non-secretor as the reference group)	-52 (-113, 9.5); p=0.096	-40 (-113, 33); p=0.273		
parity (multiparous as the reference group)	-68 (-131, -4.2); p=0.037	-48 (-121, 24); p=0.187		
